# Supplementary material for: Chitosan-Modified Biochars to Advance Research on Heavy Metal Ion Removal: Roles, Mechanism and Perspectives
Source: Materials (Basel). 2022 Sep 2;15(17):6108. doi: 10.3390/ma15176108 (PMC9457549; doi:10.3390/ma15176108)
Supplement: Supplementary file 1 [file materials-15-06108-s001.zip › materials-1826288 - supplementary.pdf]

Supplementary Material

Chitosan-Modified Biochars to Advance Research on Heavy Metal Ion Removal: Roles, Mechanism and Perspectives

Justyna Bąk[1], Peter Thomas[2], Dorota Kołodyńska[1]

[1]Department of Inorganic Chemistry, Institute of Chemical Sciences, Faculty of Chemistry, Maria Curie-Skłodowska University, Maria Curie-Skłodowska Sq. 2, 20-031, Lublin, Poland,

[2]Earthcare, LLC8524 Southport Drive, Evansville, IN 47711

(a)

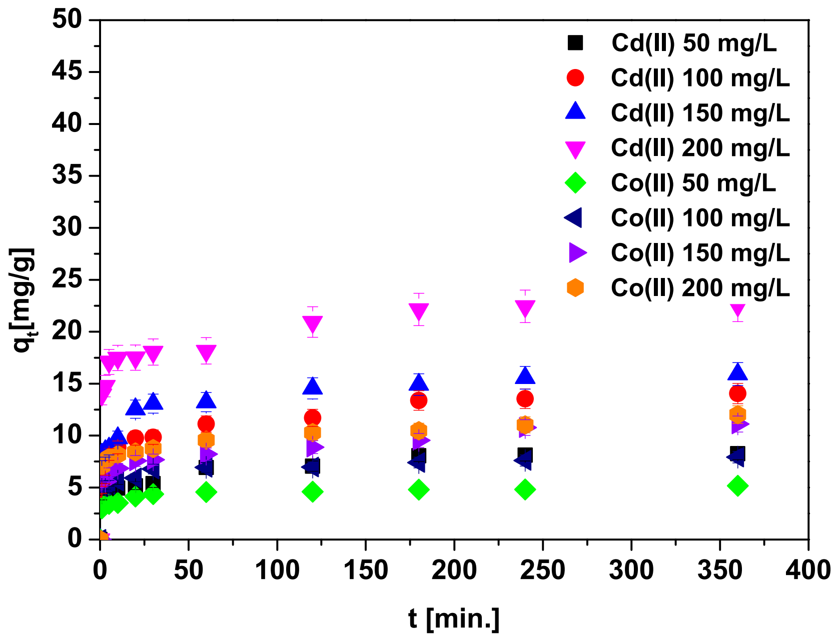

(b)

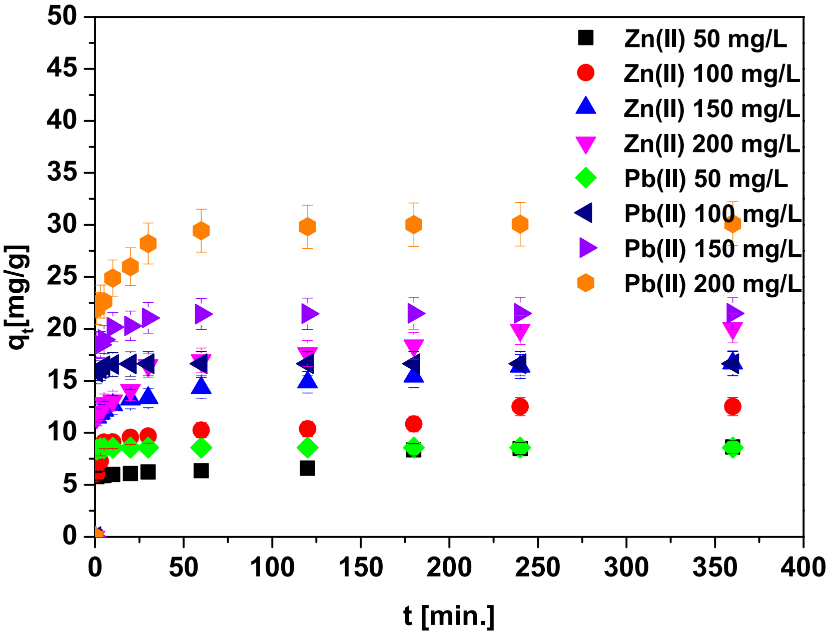
Zn(II) 50 mg/L
Zn(II) 100 mg/L
Zn(II) 150 mg/L
Zn(II) 200 mg/L
Pb(II) 50 mg/L
Pb(II) 100 mg/L
Pb(II) 150 mg/L
Pb(II) 200 mg/L
q_t[mg/g]
t [min.]

(c)

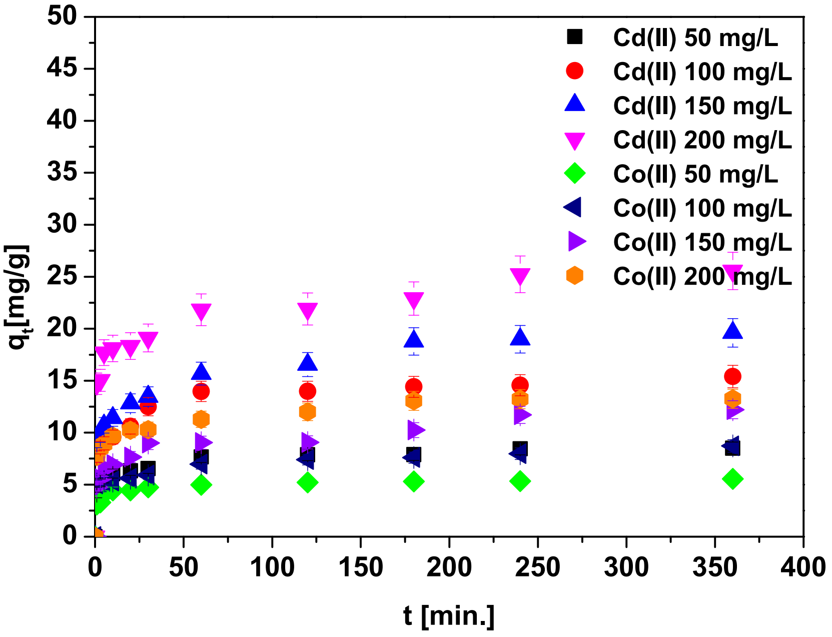
Cd(II) 50 mg/L
Cd(II) 100 mg/L
Cd(II) 150 mg/L
Cd(II) 200 mg/L
Co(II) 50 mg/L
Co(II) 100 mg/L
Co(II) 150 mg/L
Co(II) 200 mg/L
q_t[mg/g]
t [min.]

(d)

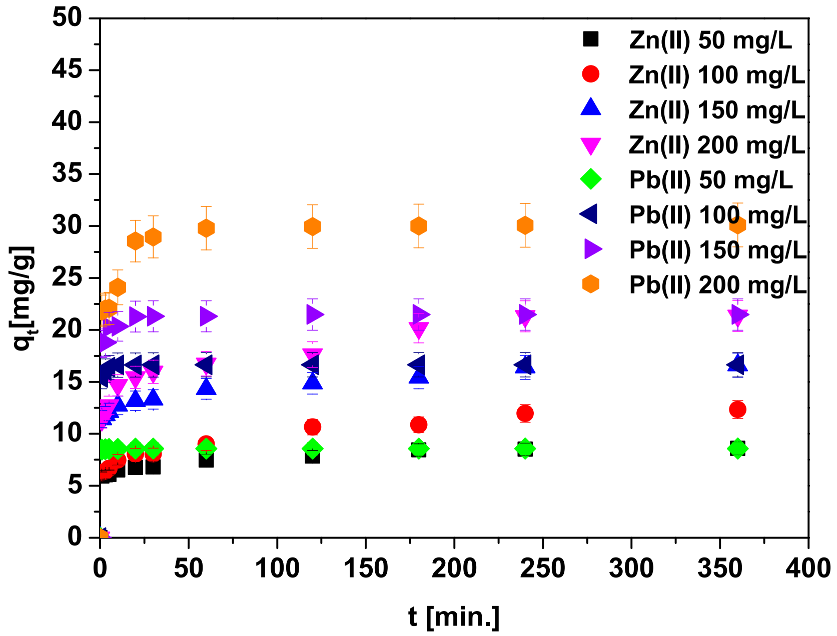
Zn(II) 50 mg/L
Zn(II) 100 mg/L
Zn(II) 150 mg/L
Zn(II) 200 mg/L
Pb(II) 50 mg/L
Pb(II) 100 mg/L
Pb(II) 150 mg/L
Pb(II) 200 mg/L
q_t[mg/g]
t [min.]
0
5
10
15
20
25
30
35
40
45
50
0
50
100
150
200
250
300
350
400

(e)

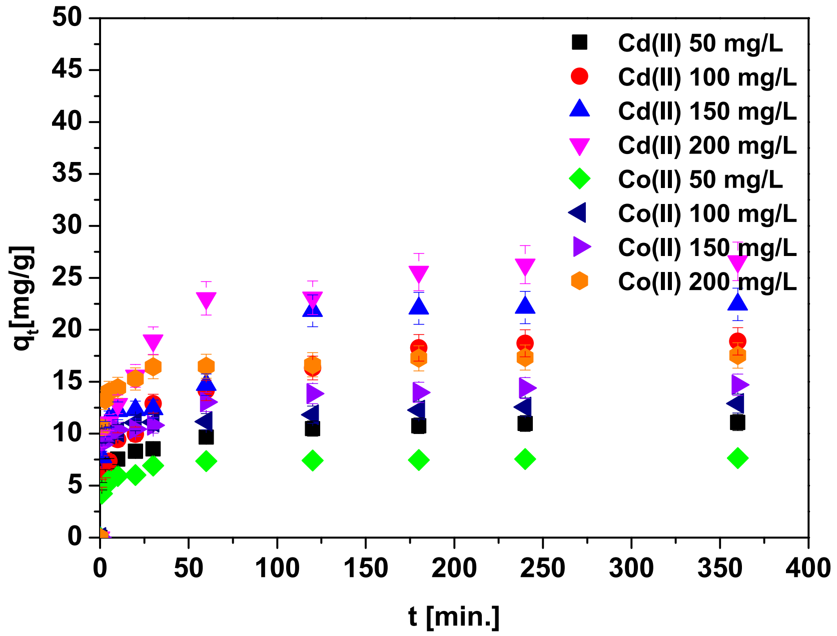
Cd(II) 50 mg/L
Cd(II) 100 mg/L
Cd(II) 150 mg/L
Cd(II) 200 mg/L
Co(II) 50 mg/L
Co(II) 100 mg/L
Co(II) 150 mg/L
Co(II) 200 mg/L
q_t[mg/g]
t [min.]
0
5
10
15
20
25
30
35
40
45
50
0
50
100
150
200
250
300
350
400

(f)

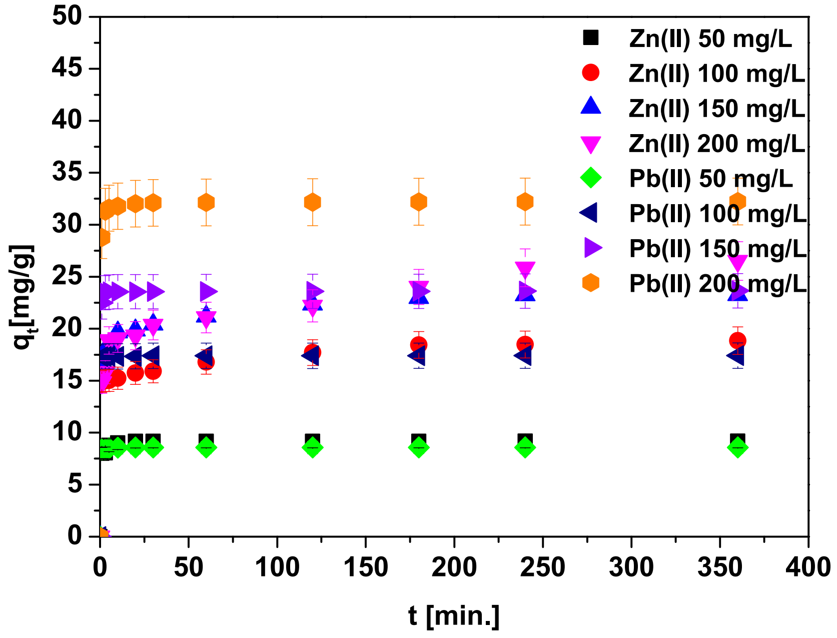

Figure S1. Effect of the phase contact time on (a) Cd(II) and Co(II) ions adsorption on BC-CS 1-1, (b) Zn(II) and Pb(II) ions adsorption on BC-CS 1-1, (c) Cd(II) and Co(II) ions adsorption on BC-CS 2-1, (d) Zn(II) and Pb(II) ions adsorption on BC-CS 2-1, (e) Cd(II) and Co(II) ions adsorption on BC-CS 4-1, (f) Zn(II) and Pb(II) ions adsorption on BC-CS 4-1 (pH 5, sorbent mass 0.1 g, shaking speed 180 rpm, temperature 293 K).

(a)

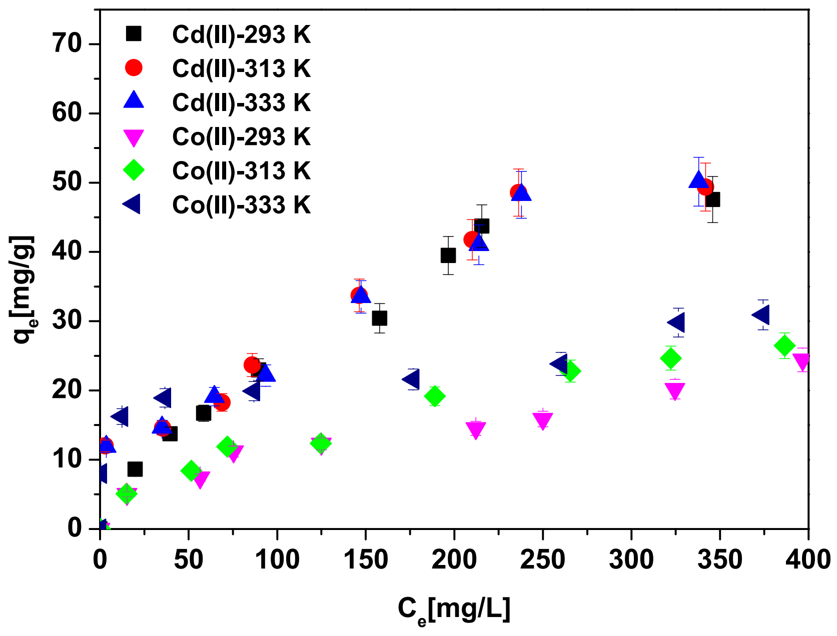

(b)

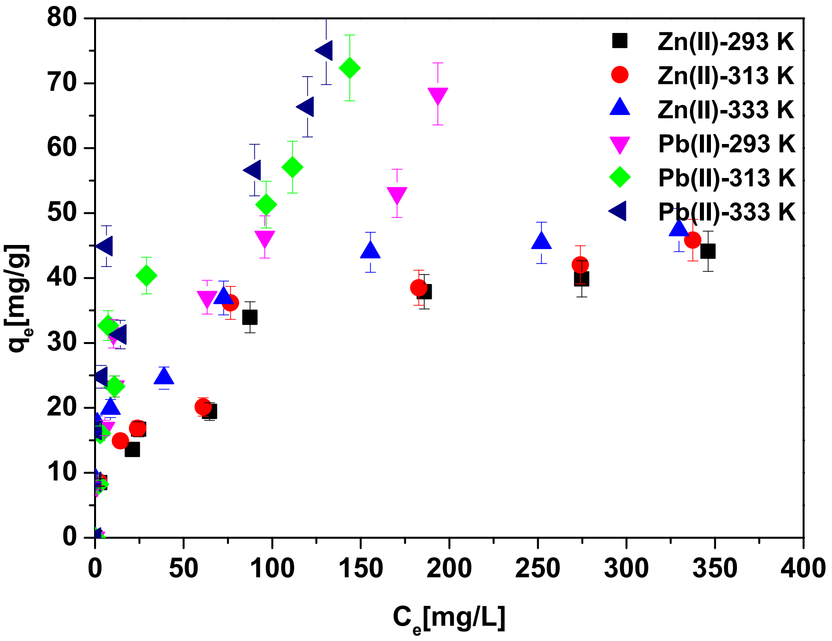

Zn(II)-293 K
Zn(II)-313 K
Zn(II)-333 K
Pb(II)-293 K
Pb(II)-313 K
Pb(II)-333 K
q_e[mg/g]
C_e[mg/L]
0
10
20
30
40
50
60
70
80
0
50
100
150
200
250
300
350
400

(c)

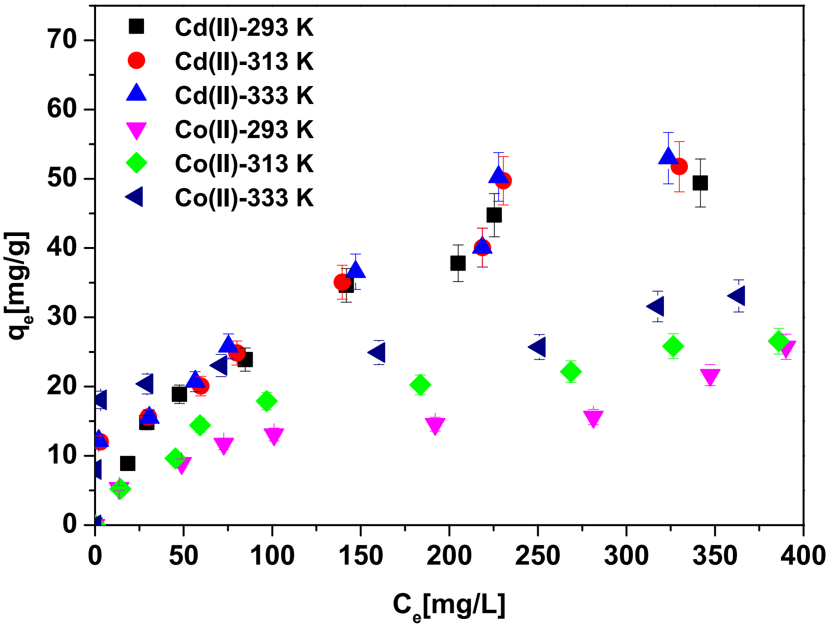

Cd(II)-293 K
Cd(II)-313 K
Cd(II)-333 K
Co(II)-293 K
Co(II)-313 K
Co(II)-333 K
q_e[mg/g]
C_e[mg/L]
0
10
20
30
40
50
60
70
0
50
100
150
200
250
300
350
400

(d)

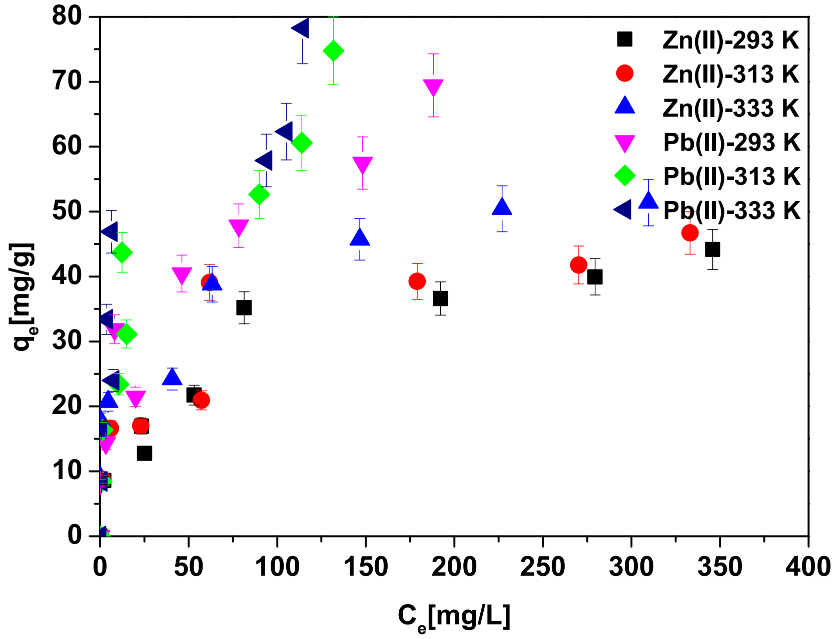
Zn(II)-293 K
Zn(II)-313 K
Zn(II)-333 K
Pb(II)-293 K
Pb(II)-313 K
Pb(II)-333 K
q_e[mg/g]
C_e[mg/L]
0
10
20
30
40
50
60
70
80
0
50
100
150
200
250
300
350
400

(e)

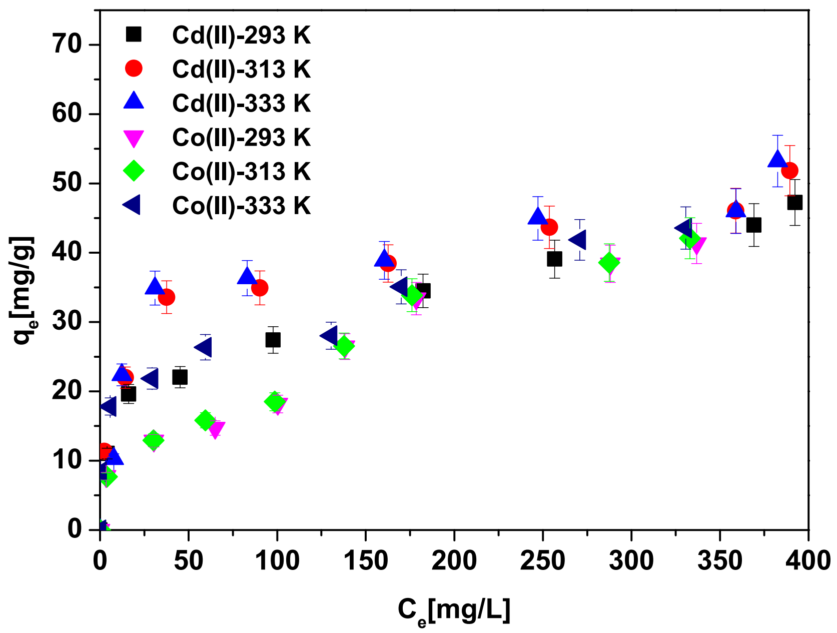
Cd(II)-293 K
Cd(II)-313 K
Cd(II)-333 K
Co(II)-293 K
Co(II)-313 K
Co(II)-333 K
q_e[mg/g]
C_e[mg/L]
0
10
20
30
40
50
60
70
0
50
100
150
200
250
300
350
400

(f)

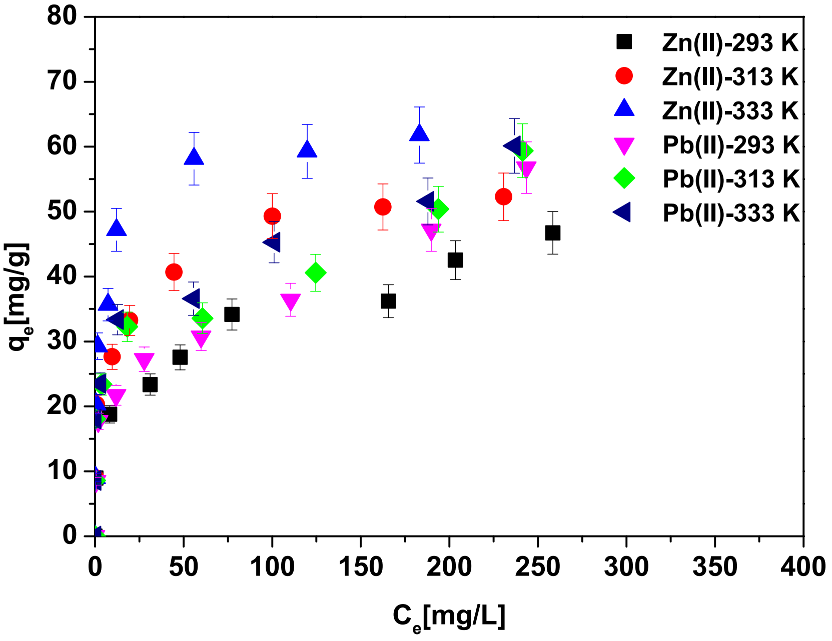

Figure S2. Effect of temperature on (a) Cd(II) and Co(II) ions adsorption on BC-CS 1-1, (b) Zn(II) and Pb(II) ions adsorption on BC-CS 1-1, (c) Cd(II) and Co(II) ions adsorption on BC-CS 2-1, (d) Zn(II) and Pb(II) ions adsorption on BC-CS 2-1, (e) Cd(II) and Co(II) ions adsorption on BC-CS 4-1, (f) Zn(II) and Pb(II) ions adsorption on BC-CS 4-1 (C_0 50-600 mg/L, sorbent mass 0.1 g, pH 5, shaking speed 180 rpm).

Table S1. Parameters for various adsorption kinetic models for Cu(II), Cd(II), Zn(II) Co(II) and Pb(II) sorption on BC-CS 2-1.

		Parameters									
		PFO			PSO				IPD		
**C_0 [mg/L]**	**q_{exp}**	$\log(q_1-q_t)=\log(q_1)-\frac{k_1 t}{2.303}$			$\frac{t}{q_t}=\frac{1}{k_2 q_2^2}+\frac{t}{q_2}$				$q_t=k_i t^{1/2}+C$		
		**q_1**	**k_1**	**R^2**	**q_2**	**k_2**	**h**	**R^2**	**k_i**	**C**	**R^2**
					**Cu(II)**						
**50**	7.95	0.78	0.018	0.821	7.96	0.146	9.229	1.000	0.010	7.761	0.753
**100**	14.01	5.86	0.010	0.875	13.93	0.009	1.814	0.996	0.217	9.881	0.915
**150**	18.22	10.00	0.013	0.930	18.36	0.005	1.748	0.996	0.339	12.029	0.719
**200**	24.53	13.40	0.011	0.972	24.68	0.003	2.102	0.995	0.328	18.347	0.987
					**Cd(II)**						
**50**	8.50	3.28	0.015	0.887	8.53	0.021	1.541	0.999	0.101	6.674	0.684
**100**	15.40	5.45	0.009	0.826	15.30	0.011	2.548	0.999	0.128	12.762	0.967
**150**	19.61	9.39	0.012	0.973	19.76	0.006	2.147	0.997	0.151	16.711	0.981
**200**	25.56	9.69	0.011	0.865	25.49	0.006	3.581	0.996	0.438	17.576	0.724
					**Zn(II)**						
**50**	8.59	2.71	0.015	0.978	8.65	0.024	1.804	0.999	0.026	8.098	0.962

**100**	12.34	5.91	0.010	0.955	12.35	0.008	1.183	0.995	0.293	7.064	0.900
**150**	16.60	4.86	0.011	0.913	16.57	0.007	3.167	0.998	0.198	12.978	0.764
**200**	21.36	10.70	0.018	0.839	21.52	0.006	2.622	0.996	0.193	17.873	0.649
					**Co(II)**						
**50**	5.56	1.48	0.009	0.855	5.53	0.045	1.371	0.999	0.047	4.659	0.933
**100**	8.72	3.39	0.007	0.959	8.55	0.013	0.941	0.994	0.151	5.724	0.963
**150**	12.21	6.08	0.009	0.866	12.06	0.007	1.004	0.988	0.331	6.101	0.831
**200**	13.26	5.40	0.019	0.957	13.39	0.014	2.439	0.999	0.033	12.653	0.763
					**Pb(II)**						
**50**	8.57	0.04	0.016	0.829	8.57	2.583	189.909	1.000	0.001	8.557	0.701
**100**	16.64	0.10	0.028	0.673	16.65	1.231	341.215	1.000	0.001	16.641	0.960
**150**	21.49	1.22	0.042	0.910	21.51	0.142	65.564	1.000	0.001	21.486	0.979
**200**	30.10	4.45	0.022	0.858	30.22	0.025	23.151	1.000	0.017	29.791	0.959

Table S2. Parameters for various adsorption kinetic models for Cu(II), Cd(II), Zn(II) Co(II) and Pb(II) sorption on BC-CS 4-1.

		**Parameters**									
		**PFO**			**PSO**				**IPD**		
**C_0 [mg/L]**	**q_{exp}**	$\log(q_1-q_t)=\log(q_1)-\frac{k_1t}{2.303}$			$\frac{t}{q_t}=\frac{1}{k_2q_2^2}+\frac{t}{q_2}$				$q_t=k_it^{1/2}+C$		
		**q_1**	**k_1**	**R^2**	**q_2**	**k_2**	**h**	**R^2**	**k_i**	**C**	**R^2**
					**Cu(II)**						
**50**	9.18	0.21	0.031	0.774	9.19	0.651	54.979	1.000	0.001	9.177	0.647
**100**	16.90	3.50	0.023	0.981	16.99	0.037	10.693	1.000	0.026	16.471	0.682
**150**	17.79	5.30	0.014	0.876	17.87	0.015	4.896	1.000	0.071	16.429	0.999
**200**	25.51	13.45	0.023	0.970	26.01	0.005	3.386	0.998	0.040	24.785	0.757
					**Cd(II)**						
**50**	11.07	4.32	0.016	0.984	11.18	0.017	2.098	0.999	0.053	10.088	0.852
**100**	18.89	12.70	0.017	0.984	19.41	0.004	1.555	0.997	0.434	11.487	0.738
**150**	22.45	13.46	0.018	0.928	23.19	0.003	1.842	0.993	0.070	21.100	0.961
**200**	26.58	15.25	0.016	0.966	27.09	0.002	2.729	0.998	0.174	23.355	0.874
					**Zn(II)**						
**50**	9.18	0.21	0.031	0.774	9.19	0.651	54.979	1.000	0.026	8.098	0.962
**100**	18.85	3.35	0.011	0.980	18.86	0.015	5.408	0.999	0.293	7.064	0.900
**150**	23.25	5.94	0.020	0.953	23.38	0.015	8.333	1.000	0.198	12.978	0.764
**200**	26.51	9.67	0.010	0.912	26.37	0.005	3.489	0.996	0.193	17.873	0.649
					**Co(II)**						
**50**	7.65	1.87	0.014	0.841	7.67	0.044	2.618	1.000	0.032	7.047	0.991
**100**	12.90	2.84	0.009	0.970	12.84	0.019	3.181	0.999	0.156	10.064	0.976
**150**	14.70	4.97	0.012	0.961	14.76	0.012	2.637	0.999	0.127	12.329	0.945
**200**	17.53	3.44	0.013	0.878	17.54	0.004	7.263	1.000	0.051	16.559	0.988
					**Pb(II)**						
**50**	8.58	0.06	0.017	0.715	8.58	2.257	166.230	1.000	0.001	8.517	0.956
**100**	17.41	0.06	0.010	0.685	17.41	1.406	426.102	1.000	0.001	17.388	0.874
**150**	23.65	0.18	0.008	0.479	23.64	0.399	222.724	1.000	0.004	23.463	0.972
**200**	32.23	0.61	0.018	0.782	32.24	0.220	229.119	1.000	0.004	32.152	0.982

Table S3. Adsorption isotherm parameters and correlation coefficients for the adsorption of Cu(II), Cd(II), Zn(II), Co(II) and Pb(II) on BC-CS 2-1.

Isotherm models	Parameters	Cu(II)	Cd(II)	Zn(II)	Co(II)	Pb(II)
**Langmuir**	$q_{e,exp}$	44.17	49.39	44.16	25.74	69.43
$q_e = \frac{q_0 K_L C_e}{1+K_L C_e}$	q_0	46.99	71.44	47.67	27.13	70.35
	K_L	0.040	0.007	0.023	0.009	0.050
	R^2	0.984	0.976	0.976	0.961	0.949
	K_F	7.44	1.72	5.86	1.87	13.29
**Freundlich**	1/n	0.327	0.609	0.344	0.405	0.296
$q_e = K_F C_e^{1/n}$	R^2	0.971	0.973	0.918	0.951	0.928
	K_T	1.425	0.086	0.646	0.143	5.498
**Temkin**	b_T	353.62	167.91	329.96	490.93	301.34
$q_e = \frac{RT}{b_T}\ln(k_T C_e)$	R^2	0.900	0.939	0.858	0.835	0.809
	q_m	0.0012	0.0030	0.0012	0.0007	0.0015
**Dubinin-**	β	0.0032	0.0073	0.0036	0.0049	0.0025
**Raduszkiewicz**	E_a	16.486	8.292	11.812	10.059	14.010
$\ln q_e = \ln q_m - \beta\varepsilon^2$ $E_a = \frac{1}{\sqrt{2\beta}}$	R^2	0.953	0.974	0.887	0.939	0.899

Table S4. Adsorption isotherm parameters and correlation coefficients for the adsorption of Cu(II), Cd(II), Zn(II), Co(II) and Pb(II) on BC-CS 4-1.

Isotherm models	Parameters	Cu(II)	Cd(II)	Zn(II)	Co(II)	Pb(II)
**Langmuir**	$q_{e,exp}$	43.24	47.26	46.72	41.34	58.76
$q_e = \frac{q_0 K_L C_e}{1+K_L C_e}$	q_0	41.67	51.63	47.07	51.51	59.02
	K_L	0.042	0.014	0.044	0.009	0.047
	R^2	0.968	0.968	0.972	0.905	0.969
	K_F	8.97	6.88	10.37	14.81	11.57
**Freundlich**	1/n	0.265	0.304	0.260	0.378	0.284
$q_e = K_F C_e^{1/n}$	R^2	0.962	0.954	0.970	0.902	0.960
	k_T	38.221	0.608	4.295	0.335	3.578
**Temkin**	b_T	628.87	332.14	424.50	337.14	332.63
$q_e = \frac{RT}{b_T}\ln(k_T C_e)$	R^2	0.740	0.854	0.904	0.761	0.909
	q_m	0.0007	0.0011	0.0010	0.0010	0.0013
**Dubinin-**	β	0.0016	0.0033	0.0025	0.0039	0.0027
**Raduszkiewicz**	E_a	17.774	12.263	14.271	11.259	16.612
$\ln q_e = \ln q_m - \beta\varepsilon^2$ $E_a = \frac{1}{\sqrt{2\beta}}$	R^2	0.818	0.922	0.971	0.846	0.954

Table S5. Thermodynamic parameters for the sorption of Cu(II), Cd(II), Zn(II), Co(II) and Pb(II) ions on: BC-CS 2-1.

Ions	K_d Temperature [K]			ΔH^o [kJ/mol]	ΔS^o [J/molK]	ΔG^o[kJ/mol] Temperature [K]		
	**293**	**313**	**333**			**293**	**313**	**333**
**Cu(II)**	0.173	0.201	0.237	6.34	7.12	-12.56	-13.91	-15.13
**Cd(II)**	0.145	0.157	0.164	2.53	-7.40	-12.12	-13.16	-14.11
**Zn(II)**	0.128	0.140	0.166	5.29	0.79	-11.81	-12.86	-14.15
**Co(II)**	0.066	0.069	0.091	6.42	-1.02	-10.21	-11.01	-12.49
**Pb(II)**	0.369	0.567	0.684	12.60	34.99	-14.40	-16.50	-18.07

Table S6. Thermodynamic parameters for the sorption of Cu(II), Cd(II), Zn(II), Co(II) and Pb(II) ions on: BC-CS 4-1.

Ions	K_d Temperature [K]			ΔH^o [kJ/mol]	ΔS^o [J/molK]	ΔG^o[kJ/mol] Temperature [K]		
	**293**	**313**	**333**			**293**	**313**	**333**
**Cu(II)**	0.222	0.557	0.601	20.52	58.55	-13.16	-16.46	-17.71
**Cd(II)**	0.121	0.133	0.139	2.94	-7.50	-11.67	-12.73	-13.66
**Zn(II)**	0.180	0.227	0.337	12.57	28.39	-12.66	-14.11	-16.12
**Co(II)**	0.123	0.126	0.132	1.41	-12.63	-11.72	-12.59	-13.51
**Pb(II)**	0.233	0.246	0.254	1.74	6.15	-13.28	-14.33	-15.33
